# Supplementary material for: A whole-animal phenotypic drug screen identifies suppressors of atherogenic lipoproteins
Source: eLife. 2026 Aug 27;14:RP105314. doi: 10.7554/eLife.105314 (PMC13521566; doi:10.7554/eLife.105314)
Supplement: Supplementary file 2 [file elife-105314-code1.zip › JHDL_primary_result/Strophanthin K/Strophanthin K_JHDL_34_015_020_2020_01_31_10_25.pdf]

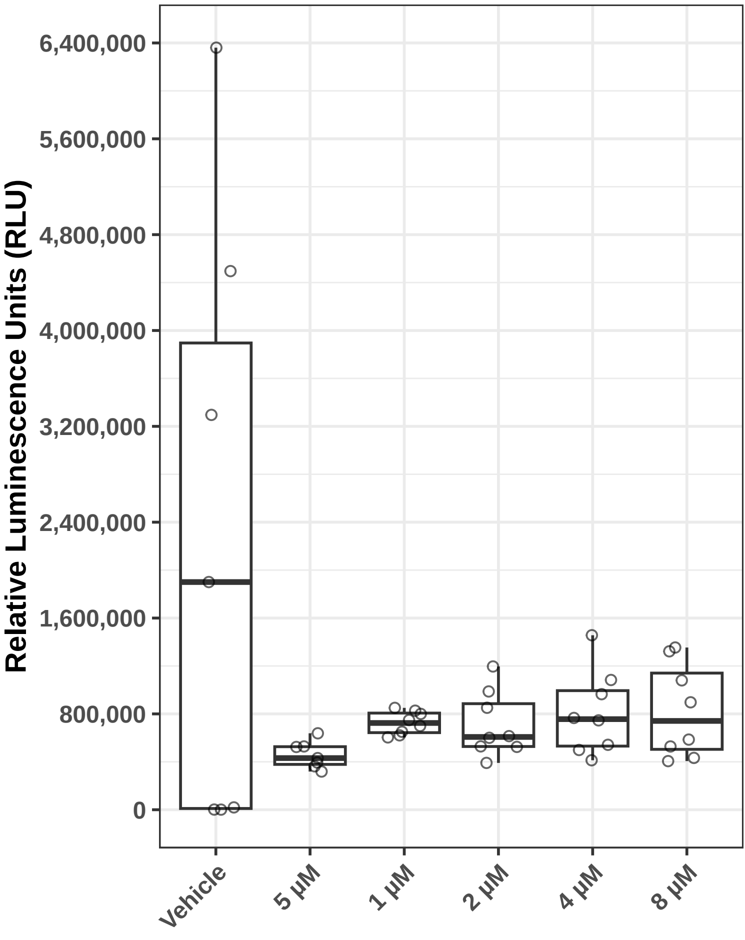

Relative Luminescence Units (RLU)
6,400,000
5,600,000
4,800,000
4,000,000
3,200,000
2,400,000
1,600,000
800,000
0
Vehicle
5 μM
1 μM
2 μM
4 μM
8 μM
